# Needs and Expectations for the myNewWay Blended Digital and Face-to-Face Psychotherapy Model of Care for Depression and Anxiety (Part 1): Participatory Design Study including People with Lived and Living Experience

**DOI:** 10.2196/69499

**Published:** 2025-06-11

**Authors:** Katarina Kikas, Kathleen O'Moore, Rosemaree Kathleen Miller, Julie-Anne Therese Matheson, Sophie Li, Kathleen Varghese, Peter Baldwin, Nicole Cockayne, Alexis Estelle Whitton, Jill Maree Newby

**Affiliations:** 1 Black Dog Institute Randwick Australia; 2 School of Psychology UNSW Sydney Kensington Australia; 3 Faculty of Medicine & Health UNSW Sydney Kensington Australia

**Keywords:** participatory design, depression, anxiety, smartphone, digital intervention, blended care, transdiagnostic

## Abstract

**Background:**

Digital mental health interventions (DMHIs) are effective in reducing symptoms of depression and anxiety. Low user engagement and uptake of DMHIs observed in previous research may be addressed by involving the intended target audience in the design of the DMHI from the outset.

**Objective:**

This study is phase 1 of a multiphase project aimed at designing, developing, and evaluating a blended DMHI for depression and anxiety in Australia. Our objective was to partner with adults with lived and living experiences of depression and anxiety on their needs and expectations of a new transdiagnostic DMHI for depression and anxiety. This included identifying strategies that would help increase their engagement with the DMHI and their preferences for integrating the DMHI with psychotherapy.

**Methods:**

A mixed methods participatory design approach was used to collect quantitative and qualitative data via a web-based survey (n=324) and semistructured interviews (n=21). Feedback was collected on participants’ needs and expectations for the DMHI, including accessibility, content, features, functionality, format, data sharing, preferred clinical support pathways, and barriers to and facilitators of user engagement. Qualitative interview data were analyzed using reflexive thematic analysis.

**Results:**

Most participants (190/257, 73.9%) preferred a DMHI delivered as a smartphone app that could be used at any time of the day. Ease of use and a well-designed interface were important, as was a positive, encouraging, and uplifting DMHI look and feel. Other preferences included symptom tracking, diverse therapeutic content, and features that facilitated social connection and peer support (eg, online community and stories of lived and living experience). Participants also suggested several strategies to enhance engagement with the DMHI, including personalization, reminders, short and achievable activities, and goal setting. Participants reported a strong interest in sharing information from their DMHI with mental health professionals (to facilitate therapy), especially regarding changes to their emotions.

**Conclusions:**

Transdiagnostic DMHIs for depression and anxiety have great potential to improve access to affordable, evidence-based mental health support. Involving people with lived and living experiences of depression and anxiety in the design, development, and conceptualization of DMHIs may improve uptake, acceptance, engagement, usability, and ultimately, treatment outcomes.

## Introduction

### Background

Globally, depression and anxiety are two of the most prevalent mental health disorders. In 2019, an estimated 280 million people were living with a depressive disorder, while >300 million people were living with an anxiety disorder [1]. Depression and anxiety commonly co-occur, with comorbidity rates as high as 50% [2]. These disorders are associated with a decreased quality of life due to poor physical health, disability, and reduced life expectancy [3], as well as lower workforce productivity and high economic costs [4]. Fortunately, there are many effective treatments for depression and anxiety that either target specific disorders or use a transdiagnostic approach [5]. However, several barriers affect access to treatment. These include high out-of-pocket costs and inconsistencies in the quality and type of care received [1]. Concerns about seeking help in person due to negative beliefs and stigma surrounding mental illness also play a role [6]. In addition, long waiting times persist due to a shortage of trained mental health professionals and inadequate funding of mental health services [1,6].

### Digital Mental Health Treatment

Digital mental health interventions (DMHIs), such as smartphone apps or online programs, address many of the barriers to face-to-face mental health care. DMHIs allow people to access psychotherapy skills and content in their own time and empower them to self-manage their symptoms [7-11]. Due to these advantages and the need to minimize face-to-face contact during the COVID-19 pandemic, many DMHIs have been developed and implemented quickly in recent years [12]. DMHIs can be used in a self-guided format (ie, without human support) [8,9], guided format (ie, with remote support from a health professional or coach), or together with face-to-face therapy sessions in person or with telehealth in a “blended” format (blended care) [12-14]. Evidence supports the efficacy of DMHIs, particularly those based on cognitive behavioral therapy (CBT), in reducing symptoms of depression and anxiety [15,16]. A 2021 meta-analysis [15] found that compared to control conditions, both guided and self-guided DMHIs significantly reduced depressive symptoms across 83 studies, with a medium pooled effect size (Hedges *g*=0.52; 95% CI 0.43-0.60). For anxiety, a recent meta-analysis [16] reported that compared to inactive controls, guided and self-guided DMHIs significantly reduced anxiety symptoms across 47 studies, with a large pooled effect size (Hedges *g*=0.80; 95% CI 0.68-0.93). While most studies have evaluated internet-delivered DMHIs (ie, delivered on websites), smartphone apps targeting depressive and anxiety symptoms have also shown positive results, with small to medium pooled effect sizes for anxiety (Hedges *g*=0.26; 95% CI 0.21-0.31) and depression (Hedges *g*=0.50; 95% CI 0.40-0.61) [17,18].

Despite this evidence showing that smartphone apps and internet-based interventions are effective in improving symptoms of depression and anxiety, DMHIs are not typically integrated into routine mental health care [19]. There are several reasons for this, including a lack of practitioner confidence and training in integrating these tools into practice, difficulties in personalizing these interventions, and a lack of integration with practice management software and electronic health records [20]. There is promising preliminary research into the combination of DMHIs with face-to-face therapy in blended care for depression and anxiety [14,21,22]. However, research on the efficacy and effectiveness of blended care is limited compared to the evidence base for self-guided and guided DMHIs [8,9,23] and is confined mainly to Europe and North America [14,24]. In practice, many existing DMHIs have high attrition rates and low user engagement, especially when accessed in stand-alone self-guided formats [25,26] and in “real-world” naturalistic settings outside of controlled research studies [27]. For example, a review by Linardon and Fuller-Tyszkiewicz [26] found that the dropout rate of users participating in studies that included smartphone apps for depressive or anxious symptoms was 36% and 21%, respectively. Dropout rates are even higher in naturalistic community settings. For instance, studies have shown that completion rates of DMHIs in naturalistic settings are at best half of what is found in clinical trials of the same intervention [28-30]. Several factors contribute to low DMHI engagement, including technical issues, motivation issues, and a lack of user-driven design [11,25,31,32]. Reviews of the existing literature also show that various features, functionality, and design considerations can promote user engagement with DMHIs. These include the perceived usefulness; positive impact and relevance of DMHIs; content that promotes good mental, physical, and cognitive health [33,34] and social connection [25,31]; design aspects (eg, ease of use and interface); personalized content and choice [35-37]; data security and privacy; and therapist guidance [25].

### Limitations of DMHIs for Depression and Anxiety

These findings provide guidance for improving participant engagement and reducing attrition for DMHIs overall, which is especially relevant given the increased availability of DMHIs following the COVID-19 pandemic. However, an often-overlooked issue is how DMHIs should be integrated into routine clinical care for depression, anxiety, and other mental disorders [9]. Many existing self-guided and guided DMHIs include linear, one-size-fits-all programs for a specific disorder (eg, major depressive disorder) or multiple disorders (eg, transdiagnostic programs for mixed anxiety or anxiety and depression). In this model, programs follow a mostly set structure, delivering a series of modules that are completed in the same order for each person. While this approach has benefits [38], the downside is that the DMHI cannot be individually tailored to suit a person’s goals, needs, symptoms, and problems. This lack of personalization negatively affects user engagement [11,25] and is a barrier for mental health professionals using these programs in practice [39]. Another important clinical consideration is that people experiencing severe depression and anxiety symptoms, and frequent suicidal ideation, use DMHIs once they are released into the community, although the programs are often not designed for these clients (eg, myCompass and THIS WAY UP) [40-42]. The needs, preferences, and expectations of people with varying symptom severity and suicidal risk levels need to be considered at the outset when developing interventions for depression and anxiety. Another factor affecting the inclusion of DMHIs in routine clinical care is that many of these interventions have been designed as “self-help” or “stand-alone” products [8,43,44]. This has contributed to the low uptake of DMHIs in practice and limits their potential use, reach, and benefits for people with depression and anxiety at various stages of care (eg, before, during, and after seeking in-person therapy).

Incorporating the needs of the intended target audience directly into DMHI design and development can potentially help address poor user engagement, high attrition, and the lack of integration of DMHIs into clinical practice. Participatory design approaches, such as co-design and co-creation, actively incorporate multiple stakeholders’ perspectives into defining a problem and collaborating with them on potential solutions [45,46]. In digital mental health, participatory design involves partnering with the intended target audience during DMHI design, development, refinement, and evaluation to ensure the product or service reflects their needs. Unfortunately, in DMHI research that uses participatory approaches, these details are often inconsistent, use varied terminology, or are not reported clearly [45,46]. Moreover, the few existing DMHI studies that use co-design principles have been limited to young adult samples (aged 18-34 years) or mental health professionals, with very few in adults aged ≥35 years [45]. Using participatory design and co-design principles to create DMHIs that fit into routine care may help adults with anxiety and depression, along with their mental health professionals, adopt blended care more easily [21]. However, to date, only 1 European study [47] reports the participatory design process during the development of a blended care intervention with people with lived experience of mental illness and psychotherapists. In that study, people with lived experience preferred a DMHI that was gentle and compassionate rather than performance based and they valued having the autonomy to choose modules themselves or have them selected by their therapist. It was also important that the DMHI included crisis support, transparent data security, and confidentiality information. Finally, people with lived experience preferred inclusive, varied, concise content delivered in various formats (eg, text, images, and videos) to ensure the DMHI was accessible to many people.

### Rationale, Objectives, and Aims

Blending digital and face-to-face therapy is a promising approach for integrating DMHIs into routine mental health care [14,21,22,24]. However, to date, only 1 study has used participatory design to incorporate the needs of people with lived experience of mental illness into the design of a blended care intervention [47]. Blended care interventions can address the limitations of DMHIs, especially stand-alone options; hence, there is a need to further investigate the utility of the blended care approach. For depression and anxiety, the option of mental health professional involvement would add an extra layer of clinical safety and permit better tailoring of DMHI use for people experiencing depression and anxiety. This study is part of a larger Australian research project that aims to design, develop, and evaluate a new transdiagnostic DMHI for adults with depression and/or anxiety (ie, myNewWay) [48]. The intervention is intended to address limitations of existing DMHIs and be suitable for use as part of blended care. Australian research on blended care is limited; however, preliminary results align with studies in other countries [14,21,22,24] and indicate that blending digital and face-to-face therapy may improve symptoms of depression [49-51] and anxiety [51]. To the best of our knowledge, there are also currently no transdiagnostic blended care models in Australia designed with target audience input and tailored to the specific needs and preferences of adults with depression and anxiety.

This study is 1 of 2 related participatory design studies in which we partnered with people with lived and living experiences of depression and anxiety and mental health professionals in the design of the new blended care DMHI. This paper reports on the findings of people with lived and living experience. Findings from mental health professionals are reported separately in part 2 of this manuscript series [52]. The DMHI will be transdiagnostic rather than diagnosis specific, as transdiagnostic approaches are acceptable, feasible, efficient, and effective for treating multiple symptoms and diagnoses within 1 treatment program [5,40]. We will use findings of this study to co-design and then develop a program that will be engaging, safe, and effective for people with varying severity levels, including those experiencing suicidal ideation. The program could be personalized to individual goals and needs and integrated into face-to-face or telehealth mental health care. We also wanted to ensure that the DMHI met the needs of adults with lived and living experience by including relevant therapeutic content and sustaining their interest with appealing design features and functionality, especially given that user engagement is associated with better treatment outcomes [15,53] and more cost-effective treatment [54,55].

Conducting participatory design within an Australian context is important to ensure that the new blended care model aligns with the needs and expectations of adults living with anxiety and depression. In Australia, adults with depression and anxiety are treated in the private mental health system and can access up to 10 psychotherapy sessions per calendar year with partial government rebates [56]. Blending DMHIs with this existing treatment pathway could increase the quality of care, and, thereby, enhance recovery by providing access to evidence-based resources for depression and anxiety before, during, and after psychotherapy. Given that most blended care research has been conducted in Europe and North America [14,24], conducting research outside these cultural contexts also helps to show which elements of blended care may be universal and which elements are not. The first aim of this study was to examine the needs and expectations of participants regarding the content, features, functionality, and look and feel of the DMHI. This also includes how people with depression and anxiety want to access the DMHI, their preferences for content delivery, and the overall blended model of care. With respect to the blended care model, we asked about the types of therapeutic guidance and human-based support they would like to receive while using a DMHI that blends with psychotherapy, as well as their preferences for sharing data from the DMHI with health professionals. Understanding participants’ preferences for including guided components within a DMHI will ensure that it meets their needs and leverages the potential benefits of guided DMHIs for depression and anxiety [15]. Our second aim was to identify factors that would affect engagement with the new blended DMHI, which could be addressed in the design of the interface, functionality, or content of the intervention to maximize sustained engagement with the DMHI.

## Methods

### Participatory Design Approach

A mixed methods convergent parallel design [57] was used for this study, which included a convenience sample of people with lived and living experience. This approach ensured that both a broad range of perspectives (ie, web-based survey) and in-depth insights (ie, qualitative interviews) were represented to achieve the study aims and conclusions [57]. Stage 1 was the web-based survey, which assessed participants’ experiences and attitudes toward DMHIs and the barriers and facilitators to their use. The survey was developed in accordance with the Checklist for Reporting Results of Internet E-Surveys [58]. For stage 2, a subset of survey respondents completed one-on-one online qualitative interviews on their preferences for the DMHI. For people with lived and living experience, stage 3 included co-design sessions and prototype testing of designs based on findings from stages 1 and 2; however, these results are not reported in this paper. The survey and interview questions were developed by the research team, which included health professionals, people with lived experience, user experience and user interface experts, and those with knowledge of implementation research (Multimedia Appendix 1). The data collection methods used in stages 1 and 2 are consistent with prior participatory design research in digital health [45]. In addition, 5 people with lived experience of depression and anxiety served as lived experience advisors during stages 1 to 3. The lived experience advisors took part in each stage of the broader project, including both the survey and qualitative interviews conducted in this study.

### Ethical Considerations

All research procedures received ethics approval from the University of New South Wales Human Research Ethics Committee (HC200541), and all participants provided informed consent before participating. If participants wished to withdraw their consent, they could contact the research team. Participant data from the online survey and interview was deidentified and replaced with a number and saved in password protected file accessible only by the research team. Participants were given the option to enter a draw to win an Aus $100 (US $65) online gift card for taking part in the online survey. For the interview, participants were reimbursed with an Aus $30 (US $20) online gift card.

### Stage 1: Web-Based Survey

The web-based 20-minute open survey was distributed between October and December 2020. The survey was advertised via the Black Dog Institute website and social media (eg, Facebook and Instagram) and the Black Dog Institute Lived Experience Resource Network. Interested participants were directed to the Black Dog Institute website, which outlined the eligibility criteria for the study and included a link to the web-based survey hosted on Qualtrics (version 2020; SAP Inc) software. At the start of the survey, respondents read the participant information sheet and consent form outlining the purpose of the research study, eligibility criteria, where their data were stored and who had access to it, the length of time to complete the study, and their reimbursement incentive. Eligibility criteria were that participants were aged >18 years, could speak English, currently lived in Australia, and had current or previous experience with anxiety or depression. The web-based survey included questions (Multimedia Appendix 1) on participant demographics, their mental health treatment history, current technology use, their experiences with DMHIs, and preferences for a new DMHI targeted at reducing symptoms of depression and anxiety. Participants were asked what features in the DMHI they would find helpful, how they would like any content displayed, and their preferred medium for accessing the DMHI (eg, smartphone and laptop). Participants’ current levels of depression, anxiety, and stress were also assessed using the validated 21-item Depression Anxiety Stress Scale (DASS-21) [59]. The DASS-21 is reported to have sound psychometric properties [60]. Respondents rate each item on a 4-point Likert scale (never=0 to almost always=3), with higher scores indicating more severe levels of depression, anxiety, or stress.

### Stage 2: Interviews

The interviews were conducted with Zoom videoconferencing software (Zoom Communications, Inc) and lasted 60 minutes on average (SD 10.52 min; range 35-74 min). The semistructured interviews included open-ended questions (Multimedia Appendix 1) that assessed attitudes toward and experiences with DMHIs, preferences for features and functionality, and suggestions for improving engagement with DMHIs. A clinical psychologist (KO) and the user experience designer (KV) facilitated interviews. All interviews were audio-recorded and later transcribed by the project officer (KK).

### Data Analysis

Quantitative data from the web-based survey were analyzed using SPSS (version 26.0; IBM Corp). Descriptive statistics were calculated for closed-ended survey questions. Sample counts and percentages were provided for each survey question. Reflexive thematic analysis was adopted for qualitative data because this methodology was appropriate for answering the research questions [61]. Following each interview, transcripts were checked and cleaned against the original audio by KK. KK (project officer), KO (clinical psychologist), and RKM (project officer) read the transcripts several times, and after familiarization, they used a general inductive approach to generate the initial set of codes. The 3 authors have experience with digital mental health and thematic analysis. Codes were categorized in Microsoft Excel (version 2024) to create the initial coding framework, which was revised iteratively by KK, RKM, and JATM (research assistant with experience in digital mental health and thematic analysis) for consensus on final themes and subthemes. Any disagreements on codes were resolved through discussion with the research team (including KO, JMN, and AEW, who are psychologists) until a consensus was reached.

## Results

### Stage 1: Web-Based Survey

#### Overview

Of the 331 adult participants who provided consent, 324 (97.9%) supplied their demographic information, and 231 (68.3%) participants completed the full survey. The demographic, mental health, and treatment characteristics of 324 participants are presented in Tables 1 and 2. The sample had an average age of 46 (SD 16.4; range 18-80) years, was predominantly female, spoke English as their first language, was born in Australia, and lived in a major Australian city. A total of 209 (68.3%) of 306 participants rated their digital literacy as good to very good, with the rest rating it as acceptable (n=65, 21.2%) or poor (n=12, 3.9%). Approximately half (156/306, 51%) of the participants spent 1 to 3 hours online daily, and >40% (131/306, 42.8%) spent >4 hours online daily. A small number of participants (19/306, 6.2%) spent <1 hour online daily.

**Table 1:** Demographic characteristics for the web-based survey (n=324).

Demographic characteristics		Participants, n (%)
**Gender**		
	Women	204 (63)
	Men	113 (34.9)
	Nonbinary	7 (2.2)
First language English		305 (94.1)
Born in Australia		244 (75.3)
Aboriginal or Torres Strait Islander origin		5 (1.5)
**Residential location in Australia^a^**		
	Major cities	221 (69.3)
	Regional or remote	98 (30.7)
**Relationship status^a^**		
	Single or never married	91 (28.5)
	In a relationship	58 (18.2)
	Married or de facto	102 (32)
	Separated, divorced, or widowed	66 (20.7)
	Other relationship status	2 (0.6)
**Level of education^a^**		
	Less than high school level	25 (7.8)
	High school level	53 (16.6)
	Certificate or diploma	133 (41.7)
	University undergraduate degree	58 (18.2)
	University postgraduate degree	41 (12.9)
	Other levels of education	9 (2.8)
**Employment status^a,b^**		
	Full-time paid work	106 (33.2)
	Part-time paid work	74 (23.2)
	Not in the labor force	59 (18.5)
	Unemployed	56 (17.6)
	At-home parent	9 (2.8)
	Carer for a family member	7 (2.2)
	Student	7 (2.2)
	Other employment status	14 (4.4)

^a^Percentages for residential location, relationship status, level of education, and employment status were calculated based on the number of participants who responded to these questions (n=319).

^b^Percentages do not add up to 100% as respondents were allowed multiple responses.

**Table 2:** Treatment and mental health characteristics for the web-based survey (n=318).

Characteristics		Participants
Current chronic condition, n (%)		126 (39.5)
Carer of a person with depression and anxiety, n (%)		58 (18.2)
**Ever experienced a mental health problem, n (%)**		
	No	6 (1.9)
	Anxiety	30 (9.4)
	Depression	34 (10.7)
	Anxiety and depression	198 (62.3)
	Not sure	7 (1.6)
	Other mental health problem	43 (13.5)
**Past mental health treatment^a^, n (%)**		
	None	50 (14.9)
	Medication	225 (67.2)
	Therapy with psychologist	233 (69.6)
	Therapy with psychiatrist	129 (38.5)
	Counseling from GP^b^	129 (38.5)
	Counseling from other MHP^c^ (ie, nurse and social worker)	99 (29.6)
	Online mental health program	63 (18.8)
	Other treatment types	30 (9)
**Current mental health treatment^a^, n (%)**		
	None	110 (32.8)
	Medication	153 (45.7)
	Therapy with psychologist	113 (33.7)
	Therapy with psychiatrist	46 (13.7)
	Counseling from GP	60 (17.9)
	Counseling from other MHP (ie, nurse and social worker)	24 (7.2)
	Online mental health program	14 (4.2)
	Other treatment type	13 (3.9)
**DASS-21 scores^d^, mean (SD)**		
	Depression	18.94 (11.50)
	Anxiety	13.60 (9.31)
	Stress	19.16 (8.63)
	Total scores	51.70 (25.77)
**DASS-21 depression ranges^d^, n (%)**		
	Normal	61 (20)
	Mild	43 (14)
	Moderate	82 (26.6)
	Severe	43 (14)
	Extremely severe	79 (25.6)
**DASS-21 anxiety ranges^d^, n (%)**		
	Normal	92 (29.9)
	Mild	18 (5.8)
	Moderate	70 (22.7)
	Severe	43 (14)
	Extremely severe	85 (27.6)
**DASS-21 stress ranges^d^, n (%)**		
	Normal	111 (36)
	Mild	47 (15.3)
	Moderate	72 (23.4)
	Severe	60 (19.5)
	Extremely severe	18 (5.8)

^a^Percentages do not add up to 100% as respondents were allowed multiple responses.

^b^GP: general practitioner.

^c^MHP: mental health professional.

^d^Results for the 21-item Depression Anxiety Stress Scale (DASS-21) were calculated based on the number of participants who responded to these questions (n=308).

#### Mental Health and Treatment

Most participants (198/318, 62.3%) had experienced both depression and anxiety (Table 2). A total of 191 (60.1%) of 318 participants were currently receiving mental health treatment, and 285 (85.3%) participants had received mental health treatment in the past. Medication was the most common form of current and past treatment, followed by therapy with a psychologist or psychiatrist (Table 2). The distribution of participants (308/324, 95.1%) who scored in the normal, mild, moderate, severe, and extremely severe ranges for the 3 DASS-21 subscales can be seen in Table 2. Approximately 80% (247/308) of participants had elevated depression scores, 70% (216/308) had elevated anxiety scores, and 64% (197/308) had elevated stress scores on the DASS-21 subscales (scored in the mild to extremely severe ranges).

#### Current and Past Use of DMHIs

Only 52 (17%) of 306 participants were currently using DMHIs, while 131 (42.8%) had used them in the past. The most commonly used DMHIs among 139 participants were mindfulness meditation apps (n=79, 56.8%), followed by CBT-based apps (n=30, 21.6%) and forums (n=13, 9.4%). The most frequently used apps were Headspace, Smiling Mind, and Calm. Of the participants who had never used DMHIs (155/306, 50.7%), the 3 most common reasons were a lack of awareness, a belief that these products are not useful or necessary, and not having found a DMHI that was helpful to them.

#### Preferences for Digital Mental Health

##### Product Promotion or Awareness

Almost 70% (177/256) of participants preferred finding out about a DMHI via a health professional recommendation, followed by social media or Google search (Table 3).

**Table 3:** Participants’ preferences for promotion and access.

			Participants, n (%)
**Finding out about the DMHI^a^ (n=256)**			
	Recommendation from a health professional	177 (69.1)	
	Social media	159 (62.1)	
	Google search	156 (60.9)	
	Recommendation from friend or family member	119 (46.5)	
	App store search	70 (27.3)	
	I am not sure	13 (5.1)	
	Other promotion pathway	10 (3.9)	
**Mode of access (n=257)**			
	Smartphone via an app	190 (73.9)	
	Laptop	85 (33.1)	
	Tablet or iPad	72 (28.1)	
	Smartphone via a web browser	55 (21.4)	
	Desktop computer	50 (19.5)	
	Other mode of access (eg, smartwatch)	11 (4.3)	
**Time of the day (n=257)**			
	Any time they felt the need to use it	164 (63.8)	
	In the evening (PM)	96 (37.4)	
	Just before bed	73 (28.4)	
	In the middle of the night (if needed)	47 (18.3)	
	In the morning (AM)	43 (16.7)	
	Upon waking up	22 (8.6)	
	Lunchtime	15 (5.8)	
	Other time of the day	6 (2.3)	

^a^DMHI: digital mental health intervention.

##### Access to Product

Most participants (190/257, 73.9%) reported wanting to access a DMHI via a smartphone app, while a third preferred (85/257, 33.1%) using a laptop (Table 3). Most participants wanted to use a DMHI when they felt the need to use it (164/257, 63.8%), in the evening (96/257, 37.4%), or just before bed (73/257, 28.4%; Table 3).

##### Willingness to Pay

Only 40 (16.5%) of the 242 participants were willing to pay for a DMHI, whereas most said maybe (n=142, 58.7%), and the rest said no (n=60, 24.8%). Of the 182 participants who responded yes or maybe, the median amount they would pay monthly for a DMHI was Aus $10.00 (US $6.5; IQR Aus $5.00-$15.00). The majority (125/176, 71%) were willing to pay between Aus $1 to $10 (US $0.65-$6.5) per month, while some participants (12/176, 6.8%) were willing to pay >Aus $50 (US $32) per month.

##### Preferred Features

**Figure 1:**
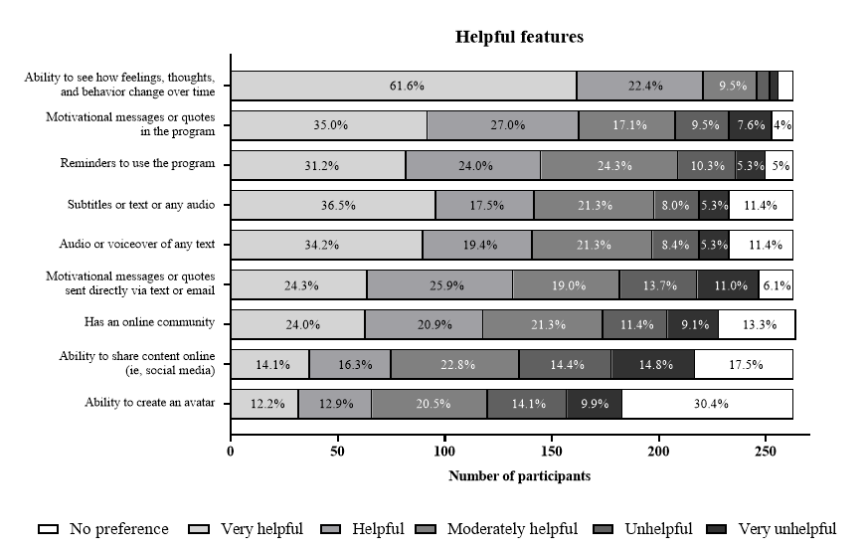
Participant preferences for the features of digital mental health interventions they find most helpful (n=263). Data labels show the frequency percentages.

Figure 1 shows which DMHI features participants considered the most helpful (ie, rated as helpful or very helpful). The most popular features were the ability to track thoughts, feelings, and behaviors over time (221/263, 84%), motivational messages (163/263, 62%), reminders (145/263, 55.1%), subtitles (142/263, 54%), and audio voiceover (141/263, 53.6%). Features viewed as moderately helpful included motivational messages sent directly to the user via email or text and the inclusion of an online community, while the least helpful features were having an avatar and the ability to share content online in general.

##### Preferred Format of Content Delivery

**Figure 2:**
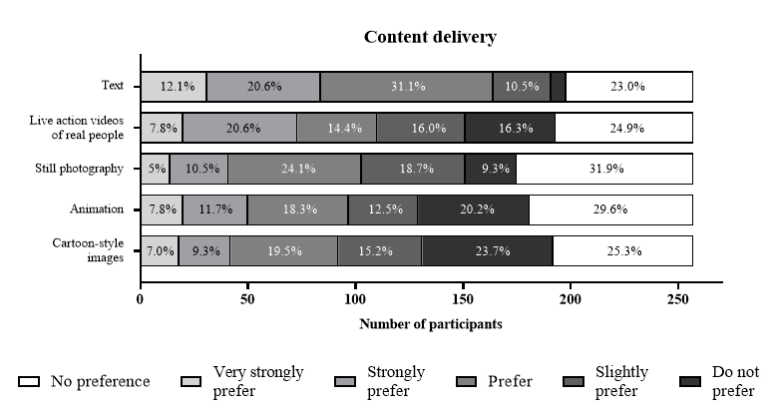
Participant preferences for preferred content delivery methods (n=257). Data labels show the frequency percentages.

Figure 2 indicates that text, still photography, and live-action videos of real people were the preferred forms of content display (ie, rated as very strongly prefer, strongly prefer, or prefer). Although there was a wide range of preferences for content display types, most participants preferred not to see information displayed in cartoon-style images (61/257, 23.7%), animation (52/257, 20.2%), or live-action videos of real people (42/257, 16.3%; Figure 2).

#### Engagement With DMHIs

Participants commonly reported that if the DMHI were effective and helped them feel better (eg, “effectiveness in making me less anxious,” “I feel it is helping me,” and “success in feeling better”), it would help them stay engaged. Some participants also suggested that being able to visualize their progress and the use of rewards, challenges, and achievement milestones would encourage users to keep coming back to the DMHI (eg, “check ins to see progress” and “rewards for logging in every day”). Participants also thought personalized and tailored content, such as “choose your own path based on your mood” and “options to turn off what you use and don’t use,” would encourage them to keep using the DMHI. Other engagement strategies included reminders and notifications (eg, “to be able to set a reminder and task to help with motivation and skills”) and having a well-designed layout (eg, “colorful,” “interactive,” “simple,” and “visual and no text blocks”).

#### Sharing Information

Participants rated psychologists, general practitioners, and psychiatrists as individuals they were most likely to share information with via a DMHI (Table 4). Participants were relatively confident in sharing information with a counselor, a friend, or a family member and less confident when sharing with a social worker, carer, or teacher. When participants were asked about what information they would like to share via the DMHI with others (ie, psychologist, friend, or teacher), most preferred to share how they were feeling over time, followed by specific program activities, whether they felt worse or better, and their goals (Table 4). Participants also rated what types of information they were most likely to share specifically with a mental health professional. The top-rated responses were how they felt over time, when they felt better or worse, their goals, and certain activities they completed within the DMHI (Table 4). Most participants (155/241, 64.3%) preferred communicating with mental health professionals via a chat box inside the DMHI, while some (86/241, 35.7%) preferred face-to-face appointments, phone calls, or SMS text messaging (Table 4).

**Table 4:** Information sharing preferences by participants for digital mental health interventions (DMHIs).

		Participants
**People I would share information with^a^, mean (SD)**		
	Psychologist	67.60 (35.81)
	General practitioner	60.33 (34.54)
	Psychiatrist	56.64 (40.20)
	Counselor	53.42 (38.39)
	Friend	52.32 (36.75)
	Family member	48.69 (36.48)
	Social worker	34.01 (36.74)
	Carer	27.45 (35.64)
	Teacher	16.48 (27.95)
**Types of content I would share^b,c^, n (%)**		
	How I am feeling over time	169 (67.9)
	Certain activities I complete in the program that I choose to share	123 (49.4)
	When I am feeling worse	119 (47.8)
	When I am feeling better	111 (44.6)
	My goals	100 (40.2)
	What I use within the program	61 (24.5)
	All the exercises and activities I complete in the program	53 (21.3)
	When I use the program	40 (16.1)
	Other types of content	19 (7.6)
**Types of content I would share with a mental health professional^a^, mean (SD)**		
	How I am feeling over time	73.71 (30.97)
	When I am feeling worse	70.42 (34.08)
	When I am feeling better	68.67 (35.05)
	My goals	64.49 (34.59)
	Certain activities I complete in the program that I choose to share	62.05 (34.75)
	What I use within the program	55.37 (36.47)
	When I use the program	52.87 (37.82)
	All the exercises and activities I complete in the program	47.92 (36.58)
**How I would communicate with a mental health professional^b,c^, n (%)**		
	Chat box inside the program	155 (64.3)
	Face-to-face appointment	102 (42.3)
	Phone call	98 (40.7)
	SMS	84 (34.9)
	Video call	43 (17.8)
	Other communication method (eg, email)	8 (3.3)

^a^Ratings were made using a 100-point scale (0=would never share and 100=extremely likely to share).

^b^Percentages for types of content (n=249) and how the participant preferred to communicate with a mental health professional (n=241) were calculated based on the number of participants who responded to these questions.

^c^Percentages do not add up to 100% as respondents were allowed multiple responses.

### Stage 2: Interviews

#### Demographic Information

A total of 21 adults participated in one-on-one qualitative interviews. Most of them (13/21, 62%) were female participants and aged 41 years on average (SD 16.4; range 21-68 years). Most spoke English as their first language (13/21, 62%), were born in Australia (11/21, 52%), and were from major Australian cities (18/21, 86%). Of the 21 participants, 14 (67%) had experienced both depression and anxiety, 3 (14%) had experienced depression only, 1 (5%) experienced anxiety only, and 3 (14%) experienced another mental health problem.

#### Thematic Analysis Findings

Eight key themes related to DMHIs were identified: (1) promotes user engagement; (2) friendly, pleasant look and feel; (3) content is multifaceted and interesting; (4) sense of connection with real people; (5) features that facilitate progress; (6) safe, trustworthy, and effective; (7) data security is important; and (8) must be low cost or free. All themes, subthemes, and illustrative quotes for each subtheme are listed in Table 5.

**Table 5:** Themes and subthemes from the thematic analysis, along with example quotes.

Themes and subthemes		Example quotes
**1. Promotes user engagement**		
	Easy to use and simple to navigate	“It just all needs to be easy. I hate learning new technology platforms, I hate it, so easy as possible.” [Participant 4]
	Personalize content and activities to user needs	“There needs to be tailored kind of options for people, different ways that they can engage with it just to keep it interesting.” [Participant 5]
	Reminders and notifications	“I really liked the fact that it gave you reminders to use it, that was great, and you could set it to what time of the day you’d like it to remind you.” [Participant 36]
	Short and achievable activities	“Maybe kind of breaking up into small tasks. So that there’s a clear plan how to start to work, whatever the user wants to achieve.” [Participant 9]
	Goal setting to support a sense of progress	“So you can set up goals...so like basically this is what I want to become or like this is what I want and it’s like a journey.” [Participant 12]
**2. Friendly, uplifting look and feel**		
	Streamlined and attractive graphic design	“It felt like it had a kind of color scheme, it looked professional.” [Participant 1]
	Warm, gentle, and inviting tone	“The app comes up and says gently to you, ‘welcome back’ or ‘welcome, glad to see you.’” [Participant 9]
	Short, positive, and encouraging messages	“And I also get like motivating notifications every day like small quotes or something, it makes me feel good.” [Participant 12]
**3. Content is multifaceted and interesting**		
	Provides options for wellness practice that support recovery	“I think things that are like mindfulness and exercise and things that can be part of anyone’s regular routine when they're recovering would be valuable.” [Participant 29]
	Broad variety of content to prevent boredom	“So variety would be really good for the app as well because yeah people if they know exactly what’s going to happen every time they do it, they’ll just get bored of it, I think.” [Participant 56]
	Program incorporates different treatment approaches	“Like ACT...and DBT as well, like, I think a lot of people connect with those better...because it seems like every single app on the market is literally all about CBT and mindfulness.” [Participant 36]
	Searchable information about mental health	“I think it would be good if there was kind of an inventory of mental illnesses that a person can look up and read the description on the symptoms and things certain things like that and maybe suggestions on what to do if you suspect that you might have it.” [Participant 1]
**4. Sense of connection with real people**		
	Personal stories shared by real people	“I think having if possible, some form of personalized stories or...there is a section there where you can read about other people who are like you and know that you’re not alone.” [Participant 63]
	Online community support	“So I think overall, the online forums I found the most, the most useful, probably because of that connection with other people with peers, basically.” [Participant 4]
**5. Features that track and aid progress**		
	Mood-tracking feature	“I liked the feature where you could input other things that are going on like how much sleep you had...you can put all that stuff and then you could sort of look and see correlations between your mood and these external factors.” [Participant 36]
	Visualize changes in mental health over time	“You can step back from it and sort of view the data on you and then, you know, work on it from there, because it just feels like it’s this evolving thing that you know comes and goes.” [Participant 51]
	Reflective journaling	“Maybe somewhere to sort of record or reflect on how far you’ve come or what you want to achieve, or like sort of like a reflection journal could be quite valuable as well.” [Participant 29]
	Share treatment progress with their psychologist	“I don't think I would use it necessarily like in (my psychologist’s) office, but maybe even just to sort of like talk about with her of like how I’m progressing, what I'm doing for my own like mental health.” [Participant 52]
**6. Safe, trustworthy, and effective**		
	Tips and activities to help feel better immediately	“If there is something that I could, if I am feeling, not necessarily in crisis, but if I am feeling not okay for any reason, that I can dip into like say a certain section and know ok, I can do this.” [Participant 5]
	Confidence in the credibility of the program	“Knowing where it’s coming from and who is being provided by.” [Participant 24]
	Access to crisis support information if needed	“There’s a kind of list of hotlines that come up that I can access at any time in case of a crisis, so I think that’s really nice.” [Participant 1]
	User-created plan when feeling unwell	“Inviting the user to list what they like to do to keep the head above water. I like to watch comedy, pursue my interest in things like that. I look to my brother, play with my pets. Yeah, having that as a list and just go alright, here’s a couple of things that you can do, but the benefit of that is that these are things you have indicated that you want to do in the past, this is your stuff.” [Participant 9]
**7. Data security and control over information sharing**		
	Control over sharing sensitive information	“Because I want to have control over that...I’ve got the utmost trust in my GP and we work out what’s going to go on. I don’t want somebody else to have knowledge or understanding of my history and come at me cold when I don't have a relationship with that person.” [Participant 19]
	Transparent information about data privacy	“Transparency is very important, and that element of trust thing comes in as well that you know you feel comfortable about dealing with an organization or service provider that you know is there for your own understanding and benefit.” [Participant 27]
	Positive about sharing data with a trusted health professional	“I know that if my psychologist found it useful that I have personally I think she’s you know, she’s a very trusted professional for me. If there is that comfort level there, I think it’s definitely like a great feature like for her to see where I’m at.” [Participant 63]
**8. Must be low cost or free**		“Yeah, I think I didn't want to pay for anything because I wasn't sure if it was going to work and to be honest, I didn't use it frequently.” [Participant 27]

#### Theme 1: Promotes User Engagement

##### Easy to Use and Simple to Navigate

Participants indicated that they wanted a DMHI that was easy to follow, intuitive, and straightforward to navigate. Suggestions included having a fast and easy sign-up process and clear navigational icons and features. Participants also mentioned negative past experiences with products that were too clunky, slow, and frustrating to navigate. This was thought to be particularly problematic for people with mental health issues who are feeling unwell or stressed.

##### Personalize Content and Activities to User Needs

Many participants emphasized personalization as a key feature for maintaining user engagement with DMHIs. Some expressed a preference for having a program that was responsive to their needs. This included the user choosing the activities or content to complete based on how they were feeling on the day and the DMHI’s ability to suggest content and activities to the user based on their interactions with the DMHI (eg, mood-tracking scores).

##### Reminders and Notifications

Participants mentioned liking reminders and notifications in apps or programs they had previously used, as this feature helped them stay on track with managing their mental health. Some preferred daily check-in reminders or notifications that could be updated at times that suited the individual’s schedule, while others valued the option to customize the content shown in the reminders and notifications.

##### Short and Achievable Activities

There was a clear preference for activities in the DMHI to be short and quick to complete. Some participants said that being able to complete a few smaller tasks would help foster a sense of accomplishment. Participants also suggested that if an activity needed to be longer, it could be divided into smaller tasks to prevent the activity from becoming too time consuming.

##### Goal Setting to Support a Sense of Progress

Participants emphasized the importance of being able to track their progress within the DMHI. Suggestions included unlocking a new stage or section in the intervention and being rewarded, offering daily challenges the user could engage in, or setting up various goals the user could work toward.

#### Theme 2: Friendly, Uplifting Look and Feel

##### Streamlined and Attractive Graphic Design

Overall, participants wanted a DMHI that was visually appealing with a professional-looking color scheme and images. However, opinions varied about the preferred colors and designs. Some participants preferred bright and vibrant colors, while others wanted soft and calming colors. Presenting information graphically (eg, pie charts and icons) was also mentioned as a desirable design feature.

##### Warm, Gentle, and Inviting Tone

The language and tone of the content within the digital product were important. They preferred the language and tone to be empathetic, calm, and encouraging. Participants also specifically mentioned that they did not want any language or tone that could be interpreted as forceful, overpowering, or aggressive. For example, some participants preferred the DMHI to have a friendly tone, akin to how a therapist would speak to them.

##### Short, Positive, and Encouraging Messages

Many participants wanted to see brief, uplifting, and inspiring messaging displayed throughout the DMHI. Participants suggested these messages could be included in notifications or shown when a user first opens the DMHI to help someone feel welcome, create a sense of connection, and validate that they made the right decision to engage with the program.

#### Theme 3: Content Is Multifaceted and Interesting

##### Provides Options for Wellness Practices That Support Recovery

Several participants wanted a DMHI that incorporated a range of wellness techniques, skills, and practices. Suggestions were diverse and included sleep hygiene, exercise guidance, spirituality resources, breathing techniques, information on healthy living, meditation exercises, and gratitude journaling. Participants wanted these components to maintain long-term recovery and sustained engagement with the DMHI.

##### Broad Variety of Content to Prevent Boredom

Participants stressed the importance of offering a wide range of up-to-date and relevant content in the DMHI that addresses the various experiences and situations that users might encounter. This breadth and variety of content would also ensure the user could always find something new and interesting, preventing boredom and sustaining their interest in the DMHI in the long term.

##### Program Incorporates Different Treatment Approaches

Many participants had used CBT-based apps and programs in the past. These participants expressed that CBT was not suitable for all individuals and wished to see other therapeutic modalities, such as dialectical behavior therapy, acceptance and commitment therapy, and mindfulness, included.

##### Searchable Information About Mental Health

Some participants reported that a DMHI should include an information section where users could search for mental health information. For example, 1 participant suggested an inventory of mental health conditions accompanied by descriptions of their symptoms. Another participant mentioned the value of a search section to access articles or advice, allowing users to search for specific topics related to their struggles.

#### Theme 4: Sense of Connection With Real People

##### Personal Stories Shared by Real People

Several participants mentioned that they valued connecting with others who were also experiencing mental health challenges. Participants suggested that these personal stories could be shared via the DMHI through video or written accounts, either by reading stories or participating in an online forum. Some participants expressed a desire to provide support to others dealing with anxiety and depression, drawing on their past positive interactions and helping others in other apps and forums.

##### Online Community Support

Some participants reported that being able to interact with an online community of other individuals facing mental health challenges would enhance their engagement with the DMHI and help reduce feelings of loneliness. However, some participants highlighted the importance of ongoing safety monitoring and moderation if an online community was included in the DMHI.

#### Theme 5: Features That Track and Aid Progress

##### Mood-Tracking Feature

Most participants preferred to monitor their mood over time using the DMHI. Some participants suggested that it would be helpful to have the ability to track other factors, such as sleep, coffee intake, and menstrual cycles, within the mood-tracking feature to observe how these variables relate to changes in mood.

##### Visualize Changes in Mental Health Over Time

Several participants preferred to view their tracking data collected by the app visually to see how their mental health changed over time. They also wanted to view their data plotted at different calendar frequencies, such as weekly, monthly, or yearly intervals.

##### Reflective Journaling

Some participants recommended including a section for recording and reflecting on achievements and progress. They believed this would be valuable for their recovery and allow them to reflect on their progress and growth.

##### Share Treatment Progress With Their Psychologist

Some participants were keen to share their data from the DMHI with their psychologist to inform them of their progress between therapy sessions and initiate discussion in their sessions.

#### Theme 6: Safe, Trustworthy, and Effective

##### Tips and Activities to Help Feel Better Immediately

Participants mentioned that it would be useful to have a section in the DMHI where users could access tips and activities to help calm them down when they were feeling overwhelmed (eg, panicking or anxious). Suggestions included easy meditations, mood-lifting activities, and breathing exercises.

##### Confidence in the Credibility of the Program

Several participants emphasized that the content of the DMHI should be backed by scientific research and evidence. Some participants also highlighted that the intervention should be developed by a reputable organization and in collaboration with mental health professionals and researchers.

##### Access to Crisis Support Information if Needed

Some participants thought it was important to include crisis support information, such as mental health hotline numbers and websites.

##### User-Created Plan When Feeling Unwell

A few participants suggested that the DMHI should offer users the option to create a plan for challenging times or when life feels overwhelming. These participants recommended that the plan include options for pursuing personal interests and listing strategies or activities that they found helpful when they felt unwell.

#### Theme 7: Data Security and Control Over Information Sharing

##### Positive About Sharing Data With a Trusted Health Professional

Many participants were comfortable sharing data from the DMHI with a trusted health professional. Participants also highlighted several benefits of sharing the data with their health professional, such as streamlining information sharing, allowing the health professional to monitor their client’s mood between sessions, and storing and sharing homework digitally with the health professional. All these benefits were thought to enhance face-to-face therapy.

##### Control Over Sharing Sensitive Information

Participants emphasized the private and personal nature of their mental health journey. Consequently, many participants thought it was important to have control over what information they could share from the DMHI and the ability to choose whom they could share it with. Participants also felt that sharing information should be an optional feature included in the DMHI and that this feature could be managed by the client.

##### Transparent Information About Data Privacy

A few participants were concerned about where the DMHI’s data would be stored and who would oversee the data. These participants emphasized that it was important for the DMHI to be transparent with this information.

#### Theme 8: Must Be Low Cost or Free

Nearly every participant interviewed expressed reluctance to pay for a mental health DMHI. Some reported that the high cost of in-person therapy would mean that the cost of an app would be an additional financial burden for people seeking mental health treatment. Other participants hesitated to pay for a DMHI without knowing whether the intervention was effective.

## Discussion

### Principal Findings

The objective of this study was to use participatory design to incorporate the perspectives of people with lived and living experiences of depression and anxiety into a new DMHI that could be integrated into clinical practice as part of a blended care model. To the best of our knowledge, this research is the first Australian study to partner with adults with living and lived experiences of depression and anxiety and use participatory design to conceive a blended care DMHI. Our mixed methods approach also provided valuable insight into our participants’ needs, preferences, and expectations of DMHIs and clarified how the new DMHI could be integrated with psychotherapy in a blended care model. Survey data highlighted how respondents wanted to access the DMHI and their preferences for features, content delivery, and sharing of information via the DMHI. Interview findings converged with survey data but also provided in-depth detail on preferred content and features and how to sustain engagement with the new DMHI. To date, the study findings have informed the designs for a new Australian blended care digital system called myNewWay [48], which includes a mental health smartphone app that can be used alone or as part of blended care.

### DMHI Needs, Preferences, and Expectations

Survey findings indicated that participants preferred accessing a DMHI via a smartphone app, rather than on a computer or tablet. This preference and other findings may reflect the opinions of people who are familiar with digital technology, as the level of digital literacy was relatively high in the study sample. Two important factors for addressing the needs, preferences, and expectations of people with lived and living experiences of depression and anxiety were ensuring that the DMHI was well designed and easy to use. Qualitative interviews also highlighted that including a variety of therapeutic content was important to people with lived and living experiences of depression and anxiety. These findings are consistent with DMHI research conducted before, during, and after the COVID-19 pandemic [11,31,47,62], indicating these 3 factors should be considered in the design of DMHIs for depression and anxiety. Though no clear trends in the graphic design of the DMHI were identified in surveys and interviews, participants wanted the overall esthetic, tone, and feel of the DMHI to be positive, encouraging, and uplifting. This is an important consideration given that past research has found that the esthetic qualities of DMHI design are linked to better usability and user-friendliness [11,62].

Participants were keen for a DMHI to be user-friendly, simple to navigate, and lacking in technical issues, given that (a lack of) ease of use was often associated with past negative DMHI experiences and disengagement. However, most participants were currently using or had used DMHIs, which aligns with the relatively high levels of digital literacy in the study sample. Future research should give special consideration to defining whether these qualities can be addressed in a similar way for people with higher or lower levels of digital literacy. In addition, a novel finding from qualitative interviews that builds upon previous work is that DMHI users preferred to receive short, positive motivational messages within the DMHI or via push notifications (if app based). This finding is aligned with research conducted before the COVID-19 pandemic [63], which found short motivational messages to be the most commonly used component of the myCompass self-help program. While this preference for short, positive motivational messages may be specific to conditions before the COVID-19 pandemic and DMHI users with high digital literacy, existing literature does suggest that the language used in DMHIs should be hopeful and confident [64]. Moreover, people with depression and anxiety may be experiencing concentration and working memory problems alongside any mental health symptoms or co-occurring issues, further supporting the notion that the length of messages should not be overly long.

In this study, the appeal of the DMHI was also connected to whether the product helped to improve their mental health symptoms and well-being. These results support the findings of the systematic review by Borghouts et al [25], who found that the perceived effectiveness of a DMHI will affect whether a user engages with it. Participants also suggested that the DMHI could help them monitor their mental health symptoms via a self-tracking feature (ie, mood and sleep), which is consistent with previous research [31,62]. One point of differentiation between this study and past research is that interview findings showed which aspects of self-tracking were most preferred, including the visualization of changes over time, reflective journaling, and the ability to share progress with a mental health professional. In qualitative interviews, participants also expressed a strong preference for diverse DMHI content that referred to wellness practices in addition to a wide range of therapeutic techniques, such as healthy eating, physical exercise, and gratitude practices. This desire for diverse and comprehensive content may have stemmed from many participants’ previous experiences with DMHIs that focus on a single therapeutic modality, such as CBT or mindfulness. Participants also wanted the DMHI to include features that help facilitate social connections, which again was most evident in the qualitative interview findings. This is unsurprising, given the importance of social connectedness in positive mental health outcomes [33], especially for depression [34]. Features that support social connectedness were also desired as an inclusion in past DMHIs [11,25], and the need for isolation during the COVID-19 pandemic may have exacerbated this need. Suggestions included incorporating an online community where DMHI users could connect with peers with similar experiences or being able to read or listen to personal stories of people who had overcome mental health challenges.

In line with our secondary aim, participants identified 4 strategies in surveys and interviews that could enhance user engagement with a DMHI: personalization, reminders, goal setting, and including short and achievable activities within the DMHI. Consistent with previous research [11,31,62], participants wanted to personalize a DMHI to suit their needs and set goals. Unlike other studies that saw personalization as customizing the interface (ie, colors, theme, and music) and reminders, qualitative interviews showed that participants viewed personalization as the ability to pick and choose the content and activities or have the DMHI suggest them based on their responses. These findings are especially important, given that a lack of DMHI personalization is a common barrier to user engagement [25] and a common source of user dissatisfaction [11]. Participants preferred a modular, flexible design that could be individually tailored over a less flexible linear program where all users received the same content. Consistent with previous research [35,36,62], participants suggested using custom reminders to engage and maintain user interest in the DMHI in both surveys and qualitative interviews. For example, by allowing users to customize when reminders are delivered or by prompting the user to check in with the DMHI throughout the day. Furthermore, the finding from qualitative interviews that short and achievable activities would facilitate engagement with a DMHI was unique, yet important to consider, as users of DMHIs may be experiencing anxiety and depression and associated symptoms, such as rumination, fatigue, and low energy, which may hinder their ability to engage with longer activities. On the basis of their systematic review, Borghouts et al [25] proposed that shorter module durations were associated with better user engagement in self-guided DMHIs. However, this finding was only based on 2 studies, suggesting that more research is needed to verify this finding.

### Integrating DMHIs Into Routine Care

The study findings provide insight into participants’ preferences regarding whether and how they would use a DMHI with health professionals in a blended model of care. However, it is important to note that these findings represent the views of people who tended to have high digital literacy and were either currently using or had used DMHIs. Participants were open to sharing their information with health professionals via the DMHI, and there was no clear preference for a DMHI to be self-guided or guided in surveys and qualitative interviews. These findings are in line with the potential need for a program to be used flexibly, either in a self-guided or guided format, throughout their mental health recovery and navigation through mental health treatment. Survey data indicated that participants were also comfortable with and positive about sharing data from a DMHI with a trusted health professional, such as a psychologist, general practitioner, or psychiatrist. These findings extend prior research by highlighting that participants wanted autonomy and control over what information they could share, which is especially relevant given the rapid acceleration of remote care via DMHIs and other digital technology during the COVID-19 pandemic [12]. Several participants expressed concerns about how the data collected by the DMHI would be used, stored, and accessed. These results align with a blended care qualitative study [65], which found that clients were not concerned about sharing personal data via an app if their data were kept secure, anonymous, and private. Previous research on user perceptions of stand-alone DMHIs [25] and blended DMHIs [47] also confirmed that data security, confidentiality, and transparency affect user uptake of DMHIs both before and during the COVID-19 pandemic.

Other factors that impacted whether someone was willing to use a DMHI were the product’s credibility, cost, the inclusion of crisis support, and immediate distress management. In qualitative interviews, participants emphasized that a DMHI should be backed by scientific research and developed by a reputable organization, consistent with previous research that showed that inadequate information about credibility can affect user trust in a DMHI [11]. The inclusion of a crisis support section was highlighted as important in this study, as well as in the European user-centered study by Behr et al [47]. The inclusion of this feature can act as an additional safety feature when the program is released into the community, especially if it is used by individuals with higher symptom severity and frequent suicidal ideation in a self-guided format. The addition of distress management tools that can help an individual feel calmer in the moment may also be helpful for people with varying severity levels and assist with developing emotion regulation and distress tolerance. Finally, in both surveys and qualitative interviews, we found that participants were reluctant to pay for a DMHI and strongly believed it should be free or offered at a very low cost (ie, Aus $1-$10 [US $0.65-$6.5]). This corresponds with existing research indicating that many studies have found that participants prefer free DMHIs [11], highlighting the need for future DMHIs to be accessed free of charge or at a low cost. Future research should explore the drivers of this low willingness to pay for DMHIs and understand whether it is due to the low perceived value of these interventions, general expectations that mental health care should be free, or other factors. Future research also needs to explore the optimal funding and business models to support the ongoing maintenance required to successfully integrate DMHIs into routine clinical care, especially given the higher availability of DMHIs overall following the COVID-19 pandemic.

### Limitations

Two main limitations may affect the generalizability of the findings of this study. The first limitation was the nature of the sample recruited for the study. Although the sample included people with a wide range of symptom severity, it predominantly consisted of female, English-speaking, and city-dwelling individuals, with most having previously received mental health treatment. Most participants had good self-rated digital literacy (229/306, 74.8%) and spent >1 hour per day online (287/306, 93.8%), and >40% (131/306) had also used DMHIs in the past. Their opinions may not be representative of the general community, who may be eventual users of DMHIs, especially those who do not usually use digital products or those who have never accessed mental health support. The second limitation was that recruitment for this study took place during the COVID-19 pandemic. Due to the need for many people to isolate before COVID-19 vaccines were widely available, the digital provision of mental health services was expedited during this time [12] (eg, telehealth). Whether the findings generalize to the mental health care system after the COVID-19 pandemic is unknown, and further research will be needed to verify this for this study and other DMHI research conducted before the COVID-19 pandemic.

### Conclusions and Future Directions

This study provides clear insights into the needs, preferences, and expectations of adults with lived and living experiences of anxiety and depression for a transdiagnostic DMHI. Although our results are most relevant for people with high levels of digital literacy and experience with DMHIs, our findings highlight the importance of using mixed methods approaches to incorporate lived experience feedback into DMHI design and development. For people comfortable with digital technology, therapeutic interventions that improve mental health symptoms and are perceived as helpful, engaging, and effective are most desirable. Specific features that could enhance user engagement for a DMHI targeted at depression and anxiety include reminders, a self-tracking feature for symptoms, and content that facilitates social connection and peer support (eg, online forum and personal stories). The DMHI for depression and anxiety should also include access to support and resources that users can access when feeling unwell or in crisis. The potential for detection of severe symptomology, such as suicidality in depression, also supports the utility of a DMHI that a person can use as part of a blended care model with a mental health professional.

To date, findings from the participatory design study have been applied in subsequent parts of the larger Australian research project [48]. Designs for the new DMHI were subjected to a co-design and prototype testing process, which was then used to develop a smartphone app that is part of the myNewWay blended care digital system. The smartphone app provides therapeutic content and symptom management strategies primarily based on CBT, including psychoeducation, cognitive restructuring, emotion awareness and acceptance, goal setting, problem-solving, behavioral activation, exposure, relaxation, and mindfulness. Results from the participatory design study with mental health professionals were used similarly and informed the development of a health professional portal [52]. Phase 2 of the larger Australian research project involved an implementation clinical trial to evaluate the myNewWay app and Health Professional portal when used alongside psychological therapy for depression and anxiety. Currently, the myNewWay blended care system is being updated based on feedback from clients and psychologists involved in the clinical trial. The blended care system will also undergo further evaluation in a randomized controlled trial to compare the effectiveness of blended care with myNewWay to the self-guided use of the smartphone app for improving symptoms of depression and anxiety.

## Appendix

Multimedia Appendix 1 Web-based survey and interview questions.

## Abbreviations

CBT: cognitive behavioral therapy
DASS-21: 21-item Depression Anxiety Stress Scale
DMHI: digital mental health intervention

## References

[1] (2022). World mental health report: transforming mental health for all. World Health Organization.

[2] Saha S, Lim CC, Cannon DL, Burton L, Bremner M, Cosgrove P, Huo Y, McGrath JJ (2021). Co-morbidity between mood and anxiety disorders: a systematic review and meta-analysis. Depress Anxiety.

[3] GBD 2019 Mental Disorders Collaborators (2022). Global, regional, and national burden of 12 mental disorders in 204 countries and territories, 1990-2019: a systematic analysis for the Global Burden of Disease Study 2019. Lancet Psychiatry.

[4] Arias D, Saxena S, Verguet S (2022). Quantifying the global burden of mental disorders and their economic value. EClinicalMedicine.

[5] Cuijpers P, Miguel C, Ciharova M, Ebert D, Harrer M, Karyotaki E (2023). Transdiagnostic treatment of depression and anxiety: a meta-analysis. Psychol Med.

[6] Moitra M, Owens S, Hailemariam M, Wilson KS, Mensa-Kwao A, Gonese G, Kamamia CK, White B, Young DM, Collins PY (2023). Global mental health: where we are and where we are going. Curr Psychiatry Rep.

[7] Newby JM, Mason E, Kladnistki N, Murphy M, Millard M, Haskelberg H, Allen A, Mahoney A (2021). Integrating internet CBT into clinical practice: a practical guide for clinicians. Clin Psychol.

[8] Weisel KK, Fuhrmann LM, Berking M, Baumeister H, Cuijpers P, Ebert DD (2019). Standalone smartphone apps for mental health-a systematic review and meta-analysis. NPJ Digit Med.

[9] Seegan PL, Miller MJ, Heliste JL, Fathi L, McGuire JF (2023). Efficacy of stand-alone digital mental health applications for anxiety and depression: a meta-analysis of randomized controlled trials. J Psychiatr Res.

[10] Paganini S, Teigelkötter W, Buntrock C, Baumeister H (2018). Economic evaluations of internet- and mobile-based interventions for the treatment and prevention of depression: a systematic review. J Affect Disord.

[11] Alqahtani F, Orji R (2020). Insights from user reviews to improve mental health apps. Health Informatics J.

[12] Lattie EG, Stiles-Shields C, Graham AK (2022). An overview of and recommendations for more accessible digital mental health services. Nat Rev Psychol.

[13] Newby JM, Upton E, Mason E, Black M (2024). Technology-based cognitive behavioral therapy interventions. Psychiatr Clin North Am.

[14] Erbe D, Eichert HC, Riper H, Ebert DD (2017). Blending face-to-face and internet-based interventions for the treatment of mental disorders in adults: systematic review. J Med Internet Res.

[15] Moshe I, Terhorst Y, Philippi P, Domhardt M, Cuijpers P, Cristea I, Pulkki-Råback L, Baumeister H, Sander LB (2021). Digital interventions for the treatment of depression: a meta-analytic review. Psychol Bull.

[16] Pauley D, Cuijpers P, Papola D, Miguel C, Karyotaki E (2023). Two decades of digital interventions for anxiety disorders: a systematic review and meta-analysis of treatment effectiveness. Psychol Med.

[17] Bae H, Shin H, Ji HG, Kwon JS, Kim H, Hur JW (2023). App-based interventions for moderate to severe depression: a systematic review and meta-analysis. JAMA Netw Open.

[18] Linardon J, Torous J, Firth J, Cuijpers P, Messer M, Fuller-Tyszkiewicz M (2024). Current evidence on the efficacy of mental health smartphone apps for symptoms of depression and anxiety. A meta-analysis of 176 randomized controlled trials. World Psychiatry.

[19] Zielasek J, Reinhardt I, Schmidt L, Gouzoulis-Mayfrank E (2022). Adapting and implementing apps for mental healthcare. Curr Psychiatry Rep.

[20] (2021). National digital mental health framework. Australian Government Department of Health and Aged Care.

[21] Buelens F, Luyten P, Claeys H, Van Assche E, Van Daele T (2023). Usage of unguided, guided, and blended care for depression offered in routine clinical care: lessons learned. Internet Interv.

[22] Owusu JT, Wang P, Wickham RE, Varra AA, Chen C, Lungu A (2022). Real-world evaluation of a large-scale blended care-cognitive behavioral therapy program for symptoms of anxiety and depression. Telemed J E Health.

[23] Lecomte T, Potvin S, Corbière M, Guay S, Samson C, Cloutier B, Francoeur A, Pennou A, Khazaal Y (2020). Mobile apps for mental health issues: meta-review of meta-analyses. JMIR Mhealth Uhealth.

[24] Ehrt-Schäfer Y, Rusmir M, Vetter J, Seifritz E, Müller M, Kleim B (2023). Feasibility, adherence, and effectiveness of blended psychotherapy for severe mental illnesses: scoping review. JMIR Ment Health.

[25] Borghouts J, Eikey E, Mark G, De Leon C, Schueller SM, Schneider M, Stadnick N, Zheng K, Mukamel D, Sorkin DH (2021). Barriers to and facilitators of user engagement with digital mental health interventions: systematic review. J Med Internet Res.

[26] Linardon J, Fuller-Tyszkiewicz M (2020). Attrition and adherence in smartphone-delivered interventions for mental health problems: a systematic and meta-analytic review. J Consult Clin Psychol.

[27] Baumel A, Edan S, Kane JM (2019). Is there a trial bias impacting user engagement with unguided e-mental health interventions? A systematic comparison of published reports and real-world usage of the same programs. Transl Behav Med.

[28] Christensen H, Griffiths KM, Korten AE, Brittliffe K, Groves C (2004). A comparison of changes in anxiety and depression symptoms of spontaneous users and trial participants of a cognitive behavior therapy website. J Med Internet Res.

[29] Fleming T, Bavin L, Lucassen M, Stasiak K, Hopkins S, Merry S (2018). Beyond the trial: systematic review of real-world uptake and engagement with digital self-help interventions for depression, low mood, or anxiety. J Med Internet Res.

[30] Sanatkar S, Baldwin PA, Huckvale K, Clarke J, Christensen H, Harvey S, Proudfoot J (2019). Using cluster analysis to explore engagement and e-attainment as emergent behavior in electronic mental health. J Med Internet Res.

[31] Chan AH, Honey ML (2022). User perceptions of mobile digital apps for mental health: acceptability and usability - an integrative review. J Psychiatr Ment Health Nurs.

[32] Torous J, Nicholas J, Larsen ME, Firth J, Christensen H (2018). Clinical review of user engagement with mental health smartphone apps: evidence, theory and improvements. Evid Based Ment Health.

[33] Holt-Lunstad J (2022). Social connection as a public health issue: the evidence and a systemic framework for prioritizing the "social" in social determinants of health. Annu Rev Public Health.

[34] De Risio L, Pettorruso M, Collevecchio R, Collacchi B, Boffa M, Santorelli M, Clerici M, Martinotti G, Zoratto F, Borgi M (2024). Staying connected: an umbrella review of meta-analyses on the push-and-pull of social connection in depression. J Affect Disord.

[35] Gan DZ, McGillivray L, Larsen ME, Torok M (2023). Promoting engagement with self-guided digital therapeutics for mental health: insights from a cross-sectional survey of end-users. J Clin Psychol.

[36] Stawarz K, Preist C, Tallon D, Wiles N, Coyle D (2018). User experience of cognitive behavioral therapy apps for depression: an analysis of app functionality and user reviews. J Med Internet Res.

[37] Saleem M, Kühne L, De Santis KK, Christianson L, Brand T, Busse H (2021). Understanding engagement strategies in digital interventions for mental health promotion: scoping review. JMIR Ment Health.

[38] Andrews G, Basu A, Cuijpers P, Craske MG, McEvoy P, English CL, Newby JM (2018). Computer therapy for the anxiety and depression disorders is effective, acceptable and practical health care: an updated meta-analysis. J Anxiety Disord.

[39] Scott S, Knott V, Finlay-Jones AL, Mancini VO (2022). Australian psychologists experiences with digital mental health: a qualitative investigation. J Technol Behav Sci.

[40] Newby JM, Twomey C, Yuan Li SS, Andrews G (2016). Transdiagnostic computerised cognitive behavioural therapy for depression and anxiety: a systematic review and meta-analysis. J Affect Disord.

[41] Proudfoot J, Clarke J, Birch MR, Whitton AE, Parker G, Manicavasagar V, Harrison V, Christensen H, Hadzi-Pavlovic D (2013). Impact of a mobile phone and web program on symptom and functional outcomes for people with mild-to-moderate depression, anxiety and stress: a randomised controlled trial. BMC Psychiatry.

[42] Newby JM, Mewton L, Williams AD, Andrews G (2014). Effectiveness of transdiagnostic internet cognitive behavioural treatment for mixed anxiety and depression in primary care. J Affect Disord.

[43] De Witte NA, Joris S, Van Assche E, Van Daele T (2021). Technological and digital interventions for mental health and wellbeing: an overview of systematic reviews. Front Digit Health.

[44] Goldberg SB, Lam SU, Simonsson O, Torous J, Sun S (2022). Mobile phone-based interventions for mental health: a systematic meta-review of 14 meta-analyses of randomized controlled trials. PLOS Digit Health.

[45] Brotherdale R, Berry K, Branitsky A, Bucci S (2024). Co-producing digital mental health interventions: a systematic review. Digit Health.

[46] Vial S, Boudhraâ S, Dumont M (2022). Human-centered design approaches in digital mental health interventions: exploratory mapping review. JMIR Ment Health.

[47] Behr S, Fenski F, Boettcher J, Knaevelsrud C, Hammelrath L, Kovacs G, Schirmer W, Petrick H, Becker P, Schaeuffele C (2024). TONI - One for all? Participatory development of a transtheoretic and transdiagnostic online intervention for blended care. Internet Interv.

[48] myNewWay®. Black Dog Institute.

[49] Jacmon J, Malouff JM, Taylor N (2010). Treatment of major depression: effectiveness of cognitive-behavioural therapy with an internet course as a central component. E J Appl Psychol.

[50] Robertson L, Smith M, Castle D, Tannenbaum D (2006). Using the internet to enhance the treatment of depression. Australas Psychiatry.

[51] Sethi S, Campbell AJ, Ellis LA (2010). The use of computerized self-help packages to treat adolescent depression and anxiety. J Technol Hum Serv.

[52] Miller RM, O'Moore K, Kikas K, Matheson JA, Whitton AE, Baldwin P, Li S, Black M, Kampel L, Cockayne N, Tuttlebee F, Fraser C, Carr V, Varghese K, Newby JM Needs and expectations for the myNewWayTM blended digital and face-to-face psychotherapy model of care for depression and anxiety (Part 2): mental health professional co-design study. JMIR Preprints.

[53] Gan DZ, McGillivray L, Han J, Christensen H, Torok M (2021). Effect of engagement with digital interventions on mental health outcomes: a systematic review and meta-analysis. Front Digit Health.

[54] Koh J, Tng GY, Hartanto A (2022). Potential and pitfalls of mobile mental health apps in traditional treatment: an umbrella review. J Pers Med.

[55] Kählke F, Buntrock C, Smit F, Ebert DD (2022). Systematic review of economic evaluations for internet- and mobile-based interventions for mental health problems. NPJ Digit Med.

[56] Mental health care and Medicare. Services Australia.

[57] Schoonenboom J, Johnson RB (2017). How to construct a mixed methods research design. Kolner Z Soz Sozpsychol.

[58] Eysenbach G (2004). Improving the quality of web surveys: the Checklist for Reporting Results of Internet E-Surveys (CHERRIES). J Med Internet Res.

[59] Lovibond PF, Lovibond SH (1995). The structure of negative emotional states: comparison of the Depression Anxiety Stress Scales (DASS) with the Beck Depression and Anxiety Inventories. Behav Res Therapy.

[60] Osman A, Wong JL, Bagge CL, Freedenthal S, Gutierrez PM, Lozano G (2012). The Depression Anxiety Stress Scales-21 (DASS-21): further examination of dimensions, scale reliability, and correlates. J Clin Psychol.

[61] Braun V, Clarke V (2019). Reflecting on reflexive thematic analysis. Qual Res Sport Exerc Health.

[62] Alqahtani F, Winn A, Orji R (2021). Co-designing a mobile app to improve mental health and well-being: focus group study. JMIR Form Res.

[63] Whitton AE, Proudfoot J, Clarke J, Birch MR, Parker G, Manicavasagar V, Hadzi-Pavlovic D (2015). Breaking open the black box: isolating the most potent features of a web and mobile phone-based intervention for depression, anxiety, and stress. JMIR Ment Health.

[64] Bakker D, Kazantzis N, Rickwood D, Rickard N (2016). Mental health smartphone apps: review and evidence-based recommendations for future developments. JMIR Ment Health.

[65] Atik E, Schückes M, Apolinário-Hagen J (2022). Patient and therapist expectations for a blended cognitive behavioral therapy program for depression: qualitative exploratory study. JMIR Ment Health.

