# Supplementary material for: Needs and Expectations for the myNewWay Blended Digital and Face-to-Face Psychotherapy Model of Care for Depression and Anxiety (Part 1): Participatory Design Study including People with Lived and Living Experience
Source: JMIR Hum Factors. 2025 Jun 11;12:e69499. doi: 10.2196/69499 (PMC12198704; doi:10.2196/69499)
Supplement: Multimedia Appendix 1 [file humanfactors_v12i1e69499_app1.docx]

This is a Multimedia Appendix to a full manuscript published in the JMIR Human Factors. For full copyright and citation information see http://dx.doi.org/10.2196/jmir.69499.

## Online survey questions

### Current use of technology and digital mental health interventions

- How would you rate your digital literacy (i.e., the ability to use digital technologies)? (Very poor, poor, acceptable, good, very good)
- About how many hours a day would you spend online? (Less than 1 hour, 1-3 hours, 4-5 hours, more than 5 hours)
- Do you use any digital mental health programs or apps (e.g., to monitor your mood or improve your mental health/wellbeing)? (Yes – in the past, yes – currently, yes – in the past and currently, no).
  1. (If no) Why not? (Free text)
- Please list your top three apps or programs that you use or have used in the past to improve/manage your mental health (with your favourite first). (Free text)

### Preferences for digital mental health

#### Pathway to user promotion

- How would you find out about an online mental health program/app? Select all that apply. Options:
  1. Google search
  2. Search app store
  3. Social media (e.g., Facebook)
  4. Recommendation from friend/family member
  5. Recommendation from health professional
  6. I’m not sure
  7. Other (please specify)

#### Access to product

- How would you prefer to access an online mental health program? Options:
  1. Smartphone via an app
  2. Smartphone via the web browser
  3. Laptop
  4. Desktop computer
  5. Tablet/iPad
  6. Other (please specify)
- What time of day are you most likely to access your program? Options:
  1. Upon waking
  2. In the morning (AM)
  3. Lunchtime
  4. In the evening (PM)
  5. Just before bed
  6. Middle of the night if I needed it
  7. Any time I feel I need to use it
  8. Other (please specify)

### Willingness to pay

- Would you be willing to pay for an online mental health program or app? (Yes, maybe, no)
- How much would you be willing to pay for an online mental health program/app (dollars per month)? (Free text)

### Preferred features

- How helpful do you think the following features would be in an online mental health programs (i.e., how do you want it to work)? (rated on 6-point scale 0 = no preference, 1=very unhelpful to 5 ‘very helpful) Options:
  1. Ability to create an avatar (or online character of myself)
  2. Audio/voiceover of any text so I can listen rather than read text
  3. Subtitles/text of any audio (e.g., in videos) so I can complete program with no sound
  4. Sends me reminders to use the program
  5. Motivational messages/quotes in the program
  6. Motivational messages/quotes sent to me (e.g., text/email)
  7. Ability to see how my feelings, thoughts, or behaviours change over time
  8. Ability to share content online (e.g., social media, online forums)
  9. Has an online community

### Preferred format of content delivery

- How would you prefer the program content to be displayed? (rated on a 7-point scale 1= prefer not, 2 = no preference/don’t mind, 3 = slightly prefer, 4 = prefer, 5 = strongly prefer, 6 = very strongly prefer, 7 = NA). Options:
  1. Text
  2. Live-action videos of real people
  3. Still photographs
  4. Cartoon style images
  5. Animation
  6. Other (please specify)

### Engagement with digital mental health

#### Facilitators of engagement

- What would make you keep coming back to use the program? (Free text)

### Sharing information

- If you could share information in your program with someone who would you want to share it with? (rate acceptability on slider from 0 = ‘never’ to 100 ‘extremely’) Options:
  1. A friend
  2. Family member
  3. Carer
  4. Teacher
  5. Social worker
  6. Counsellor
  7. General practitioner
  8. Psychologist
  9. Psychiatrist
- If you could share information in your program, what would you want to share? Let person know:
  1. When I use the program
  2. What I use within the program
  3. How I am feeling over time
  4. When I am feeling worse
  5. When I am feeling better
  6. My goals
  7. All the activities/exercises I complete in the program
  8. Certain activities/exercises I complete in the program that I choose to share
  9. Other (please specify)
- How likely would you be to share the following information with a mental health professional? (e.g., psychologist/psychiatrist) (rate on slider from 0 = ‘never’ to 100 ‘extremely likely’) Options:
  1. When I use the program
  2. What I use within the program
  3. How I am feeling over time
  4. When I am feeling worse
  5. When I am feeling better
  6. Goals I want to achieve
  7. All the activities I complete in the program
  8. Certain activities I choose to share
  9. Other (please specify if applicable)
- If you needed more support when you were doing the program, how would you want to communicate with a mental health professional? Options:
  1. Chat box inside program
  2. SMS
  3. Phone call
  4. Video call
  5. Face-to-face appointment
  6. Other (please specify)

## Qualitative interview questions

1. What is your main goal when using a digital mental health product?
2. {If yes} Can you tell me when it first crossed your mind that you could use a website or app to support your mental health?
   1. *Which online programs/apps do you use the most (like the best) when it comes to improving your mental wellbeing?*
   2. *{If No} Why is that? What would make you use an online program/app for your mental wellbeing?* *Do you have any favourite apps/online programs that you use regularly?*
3. Why did you choose to use it? Why did you choose it from other options?
   1. *Why did you choose to use it? Why did you choose it from other options?*
   2. *What do you like about it? (What worked?)*
   3. *What do you not like about it?*
   4. *When in your day do you use it? And how do you use it in your day to day life?*
   5. *What are the most important features for you in these products? Is there anything you look for?*
   6. *What makes you come back to using it?*
4. When you first considered using a digital program, did you have any concerns? Or do you have any concerns about digital programs? (if haven’t used)
   1. *What’s the biggest challenge for you when it comes to using digital products to improve or maintain your mental health?*
   2. *What are you currently doing to make this easier for you?*
   3. *Have you had negative experiences with mental health programs or apps?*
   4. *Have you ever been in the process of signing up or registering for an online program and then decided not to?*
   5. *Can you tell me about a mental health program or app that you stopped using? What happened? Why did you stop? What should have happened?*
5. Can you tell me about a time when you had a really great experience with a digital mental health product?
6. How do you currently go about improving or maintaining your mental health? And how much time do you typically spend on this?
7. Imagine you were now using a digital program to support your mental wellbeing. When you log-in, what is the first thing you might want to see?
   1. *How could the program help you? What other content or features do you think might be useful?*
   2. *Is there anything you would like to be sent to you without having to log-in to the program?*
8. What would keep you using the program long term?
   1. *What do you think is missing in the digital mental health products you use?*
   2. *Imagine you had a magic wand and could do anything; what would you change in the digital mental health products that you use?*
9. Would you use a mental health program if you were also seeing a health professional? Why/why not?
   1. *If yes, how would you like to use an online program along with seeing a health professional?*
   2. *Which health professional/s would you be comfortable sharing information with? Prompt: GP, psychologist, psychiatrist*
   3. *What kinds of information would you share with each of these?*
10. In addition, to a mental health professional, would you share information with anyone else?
11. What do you think is the best way to promote an online mental health program so that the people who need it can easily find it?
    1. *Have you ever recommended an online program or app to someone? How did you that?*
12. How do you feel in general about online programs or apps for mental health?
    1. *Do you believe online therapy programs can help adults experiencing depression and/or anxiety?*
13. Did you have any other comments or feedback you would like to share with us? What haven’t we asked you today that you think might be valuable for us to know?
